# Expressing Parasitoid Venom Protein VRF1 in an Entomopathogen *Beauveria bassiana* Enhances Virulence toward Cotton Bollworm *Helicoverpa armigera*

**DOI:** 10.1128/aem.00705-23

**Published:** 2023-06-05

**Authors:** Shuocheng Zeng, Zhe Lin, Xianhao Yu, Junjie Zhang, Zhen Zou

**Affiliations:** a State Key Laboratory of Integrated Management of Pest Insects and Rodents, Institute of Zoology, Chinese Academy of Sciences, Beijing, China; b CAS Center for Excellence in Biotic Interactions, University of Chinese Academy of Sciences, Beijing, China; c Engineering Research Center of Natural Enemies, Institute of Biological Control, Jilin Agricultural University, Changchun, Jilin, China; University of Queensland

**Keywords:** entomopathogenic fungi, parasitoid wasp, venom protein, immunosuppression, virulence

## Abstract

Despite entomopathogenic fungi being used in various insect pest control, it is recognized that they could replace more chemical insecticides if they were more efficient. We have found that cotton bollworm Helicoverpa armigera responded to the infection of entomopathogenic fungus Beauveria bassiana by activating the Toll pathway. Koinobiont wasps also regulate host immunity and development to ensure the survival of their progeny. Previously, venom protein VRF1 was identified in Microplitis mediator. It enters *H. armigera* hemocytes, suppresses the expression of antimicrobial peptides (AMPs) by inhibiting the Toll pathway, and prevents parasite offspring from being encapsulated. With this in mind, we thought that it might be feasible to increase the virulence of B. bassiana by embedding *VRF1* into its genome. Compared with that of wild-type (WT) B. bassiana, the median lethal dose (LD_50_) of the transformant expressing VRF1 (named BbVRF1) decreased approximately 2.36-fold, and the median time to lethality (LT_50_) was shortened to 84% when infecting *H. armigera* (a natural host of *M. mediator*). The AMP expression level of hemocytes in *H. armigera* infected with BbVRF1 strain was significantly downregulated compared to that in the control group infected with the WT. In addition, the LD_50_ of BbVRF1 against the fall armyworm Spodoptera frugiperda (an unnatural host of *M. mediator*) was decreased 3.45-fold and the LT_50_ was shortened to 73%, showing a greater virulence. Our research indicated that BbVRF1, an engineered strain of B. bassiana, has greater efficacy against pest insects both within and outside its host range (*M. mediator*), expanding the utilization of parasitoid wasp virulence effectors.

**IMPORTANCE** Mycoinsecticides are essential for the development of integrated pest management as substitutes to chemical insecticides, but their usage is limited by their inferior virulence. Thus, genetically engineered bioinsecticides, including recombinant entomopathogenic fungi, have been regarded as a breakthrough to rapidly control pests. Deep knowledge of parasitoid wasps allows us to take advantage of this natural enemy of pest insects beyond raising them for field release. Our transformant BbVRF1 (Beauveria bassiana integrated with a venom protein VRF1 from Microplitis mediator) showed a higher virulence in *H. armigera* and *S. frugiperda*, demonstrating its potential for managing natural or unnatural hosts of *M. mediator*. This result provides a new strategy regarding which venom protein of parasitoid wasps can become part of the arsenal with which to equip entomopathogenic fungi. Utilizing parasitoid wasps with this approach could easily overcome the difficulties of artificial culture and enhance the virulence of other biocontrol agents.

## INTRODUCTION

Many researchers are dedicated to using genetic engineering technology to improve the efficacy of entomopathogenic fungi, which are biopesticides well-suited as alternatives and supplements to chemical insecticides for sustainable agriculture (1). Until now, potential strains have been engineered by overexpressing endogenous genes to benefit fungal infection (such as *Pr1A*, *Bbchit1*, and *CDEP1*) (2–4) and exogenous virulence effectors for fast killing (such as *AaIT1*, *Vip3A*, *BiVSP*, *Spn43Ac*, and *Aea-TMOF*) (5–9). In addition, environmental stresses, such as unsuitable temperature, humidity, and particular wavelengths of UV light, influence infection. Genetic engineering has been used to increase strain tolerance to these environmental factors in an attempt to achieve more stable performance and longer durations in complicated application environments (10, 11). For example, the overexpression of *MrHSP25* conferred thermotolerance in Metarhizium robertsii (12), and the introduced *AfTry* and DHN-melanin synthesis genes improved UV tolerance in Metarhizium anisopliae and Beauveria bassiana (13, 14). For a stronger effect, some researchers chose to create artificially modified genes by blending and coordinating related pathogenic functional domains of different genes. The hybrid CDEP1:Bbchit1 containing a Pr1A-like protease CDEP1 and the chitinase Bbchit1 enhanced fungal virulence to a higher level than the introduction of either gene alone (3). With more genome data on fungi, pest insects, and natural enemies available, there are many more virulence genes as a resource library for transgenic fungi, placing us one step ahead of insect pest resistance.

Entomopathogenic fungi have been used as pesticides for controlling economically important insect pests for almost 200 years (15). B. bassiana and M. anisopliae are the most commercially successful strains and have been isolated from stiff-bodied insects in both temperate and tropical zones (15). In contrast to *M. anisopliae*, B. bassiana has a very broad host range, from major insect orders to ticks and mites (16). Research on B. bassiana has grown quickly over the past few years from application as biological control agents to the mechanisms of their growth and virulence. Under natural conditions, B. bassiana uses an opportunistic infection strategy, spreading its aerial conidia to adhere to any location on the host cuticle by passive events (1); this strategy has a better chance to enter the host body than those used by other microbial pathogens which require a specific pathway, such as ingestion. After adhesion, aerial conidia germinate germ tubes which penetrate the cuticle by enzymatic degradation and mechanical pressure (2, 17, 18), and then hyphae grow through the host cuticle and invade the hemocoel, where fungi proliferate by blastospores (19). Activated by as-yet unknown signals, B. bassiana blastospores grow as mycelia along the inside of the integument and breach fragile tissues, such as spiracles and the internodes of segments. Finally, aerial mycelia sporulate on the stiff body (such as in Bombys batryticatus) and begin the next life cycle (16). During B. bassiana infection, insects mainly rely on cuticular barriers, cellular immunity, and humoral immunity to defend against and remove this microbial pathogen (20). Once the hyphae penetrate the cuticles, hemocytes rapidly increase in number, immediately responding to the invasion by melanization, phagocytosis, and encapsulation. Along with systematic infection, the induced Toll pathway upregulates the expression of multiple antimicrobial peptides (AMPs) against B. bassiana (21, 22). It has been reported that Helicoverpa armigera upregulates several lysozymes (lysozymes 2, 3, and 4) and AMPs (gallerimycin, gloverin-1, and moricin-4) in the fat body and others (lysozyme 1, ceropin-7, defensin, moricin-1, and ceropin-5) in the hemocoel to combat B. bassiana (22). These effectors play key roles in the interactions between insects and fungal pathogens (23).

Parasitoid wasps, another important biocontrol agent, have evolved a series of mechanisms to manipulate host development and immunity for their progeny (24). As a koinobiont endoparasitoid with a broad host range that includes *H. armigera*, Microplitis mediator lays an egg in its lepidopterous larval host. The offspring hatches in the host body and grows with the host until it breaches the body wall, cocooning itself and waiting for eclosion. Compared with that between B. bassiana and *H. armigera*, the relationship between *M. mediator* and *H. armigera* may be more sophisticated because of the wasp’s well-equipped parasitism arsenal, which includes venom, polyDNAvirus, teratocytes, and larval secretions (25). After oviposition, these efforts function in different stages: venom (the main components are proteins) directly acts on humoral proteins, hemocytes, and the fat body to temporarily suppress host immunity (26, 27); about 2 h later, the polyDNAvirus completely infects host immune cells and begins expressing proteins and microRNAs to disrupt the defense against the wasp offspring (28); after the wasp egg hatches, the serosa/extraembryonic membrane separates into single cells (teratocytes) which persistently regulate the host (29, 30); and, during larval development, secretions of larval salivary glands and anal vesicles continually affect host physiology (25, 31–33). Although dissecting and collecting these wasp products for research is complicated, venom is relatively simple to obtain because it is collected in the venom reservoir. The components of *M. mediator* venom have been determined through integrated transcriptomic and proteomic analysis (34), and it was previously determined that VRF1 (a venom protein of *M. mediator*) cleaves Dorsal (an NF-κB factor of *H. armigera*) to suppress the Toll pathway. VRF1 is an M12B proteinase that plays an important role in preventing the recruitment of hemocytes for encapsulation and suppressing the expression of AMPs by suppressing the function of Dorsal (35). Proteins belonging to the metalloproteases are involved in various biochemical and physiological processes, and M12B (a subfamily of metalloprotease) proteases are typically found in parasitoid wasp venom, but their functions have yet to be fully elucidated (25, 34, 36–43).

Our previous work determined that B. bassiana induces the Toll pathway, which is restrained by the M12B metalloprotease VRF1. This coincidence inspired us to consider that B. bassiana, the biocontrol agent of *H. armigera*, and VRF1, a venom protein derived from its natural enemy, might be a perfect match for pest killing: VRF1 could be used to suppress the Toll pathway, which would defend the fungi infection, to increase killing speed (22, 35). In this study, a B. bassiana strain was engineered to produce the *M. mediator* venom protein VRF1, which entered host cells to modulate the Toll pathway, decreasing the median lethal dose (LD_50_) by 2.36-fold and the median time to lethality (LT_50_) to 84% for *H. armigera*. In addition, against Spodoptera frugiperda (a noctuid species related to *H. armigera* which is an invasive species outside the *M. mediator* host range), the LD_50_ of B. bassiana with VRF1 decreased by 3.45-fold, and the LT_50_ was decreased to 73%, showing a greater improvement in virulence. The results are discussed within the context of utilizing VRF1 to increase the efficacy of B. bassiana.

## RESULTS

### Expression of venom protein VRF1 in *Beauveria bassiana*.

**FIG 1:**
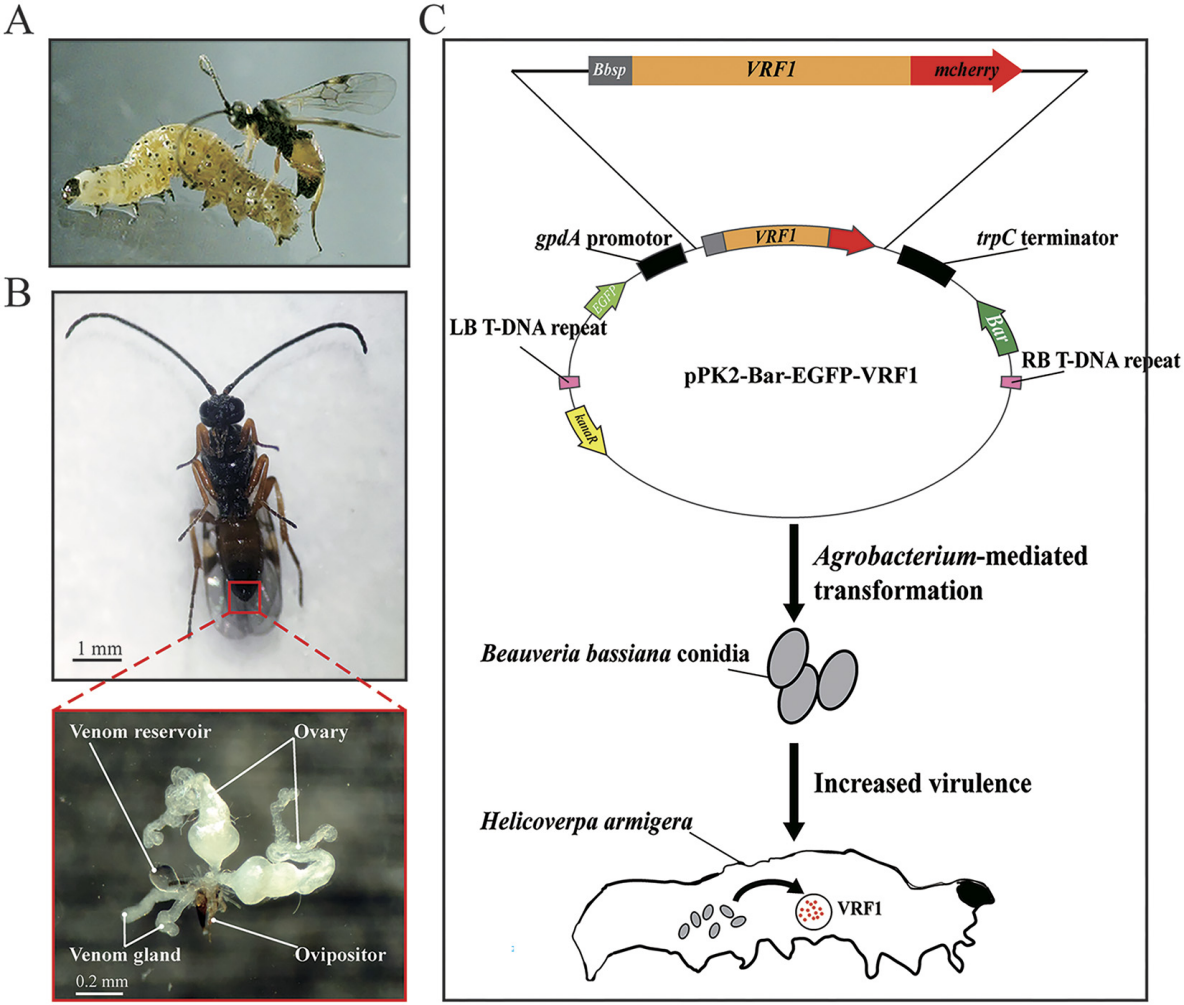
Utilizing VRF1 for fungal transformation. (A) The parasitoid wasp Microplitis mediator and its host Helicoverpa armigera. (B) Ventral appearance of *M. mediator* and anatomy of organs for parasitic behavior. Venom protein VRF1 is produced by the venom gland and accumulated by the venom reservoir. (C) Schematic diagram of the construction of Beauveria bassiana integrated with VRF1. The signal peptide of *VRF1* was replaced by the signal peptide of chitinase (named *Bbsp*) of B. bassiana ARSEF 2860 (GenBank ID: XM_008601414.1) for secretion expression, and the 3′ end of *VRF1* was fused with *mCherry* for visualization. This fusion form was ligated into pPK2-Bar-EGFP, a vector for *Agrobacterium*-mediated transformation, which retained the phosphinothricin resistance gene (*bar*) for selection. During the infection with transformant BbVRF1, VRF1 was secreted into the hemocoel of *H. armigera*.

VRF1 is a venom protein of *M. mediator*, a parasitoid wasp of the larval *H. armigera* (Fig. 1A). VFR1 is expressed in the venom gland and accumulates in the venom reservoir (Fig. 1B). To express VRF1 in the fungus, we used a modified *Agrobacterium* vector pPK2-Bar-EGFP, which was derived from the skeleton plasmid pPK2 (2), for transformation of B. bassiana. We inserted *VRF1* into the vector with modifications. We replaced the VRF1 signal peptide with the signal peptide of chitinase of B. bassiana ARSEF 2860 (GenBank ID: XM_008601414.1) for VRF1 secretion in B. bassiana. For fluorescence localization of VRF1 in living cells, *mCherry* was fused to the 3′ terminus of *VRF1*. The schematic diagram is displayed in Fig. 1C. The conidia of B. bassiana ARSEF 2860 were harvested from a potato dextrose agar (PDA) culture and cocultured with Agrobacterium tumefaciens AGL1 transformed with pPK2-Bar-EGFP-VRF1 on induction medium agar plates for A. tumefaciens-mediated transformation.

**FIG 2:**
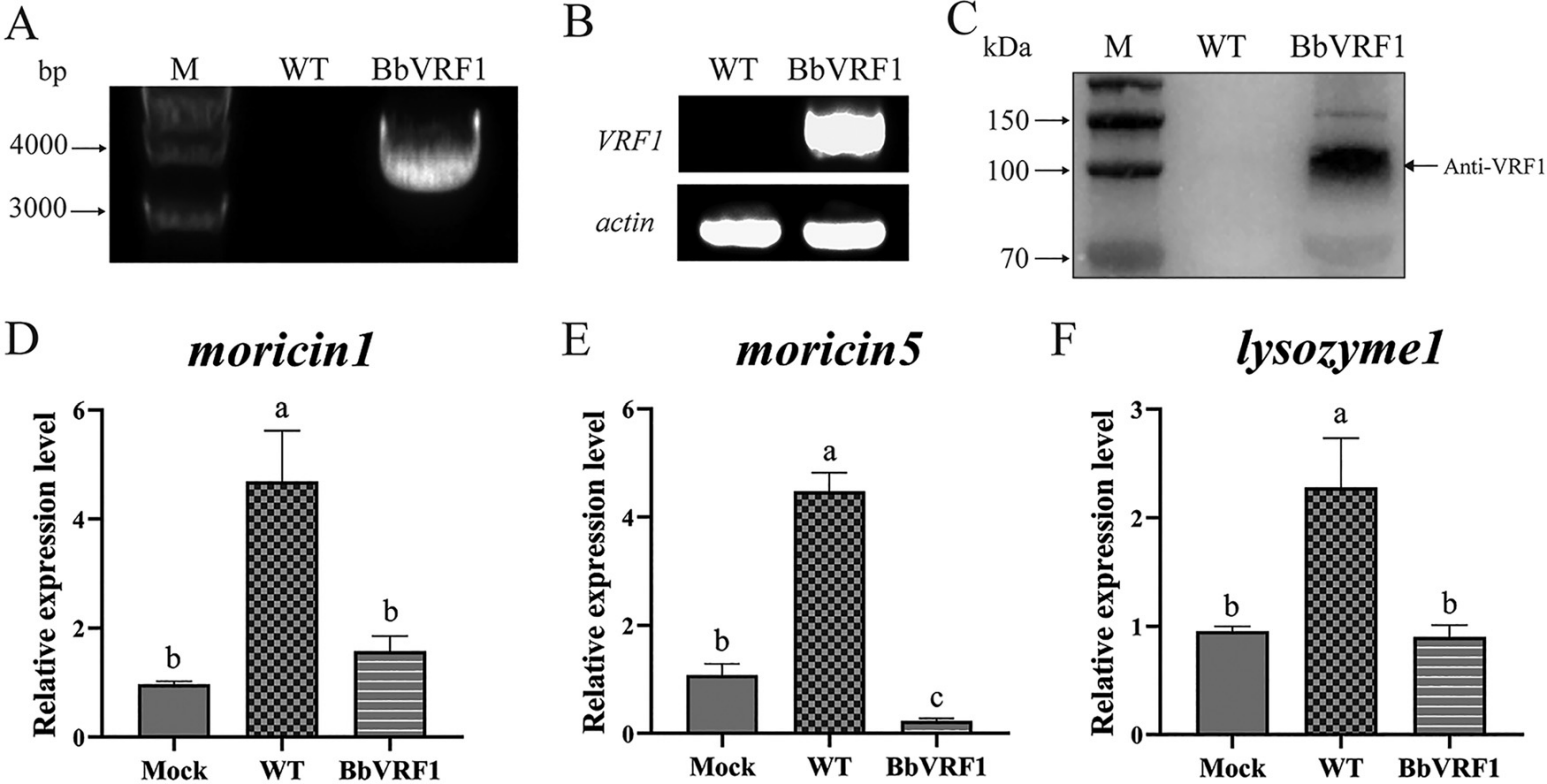
Expression of BbVRF1 and AMP expression levels after infection. (A) PCR detection for the presence of the fragment (cassette *gpdA* promoter + *Bbsp* + *VRF1* + *mCherry* + *trpC* terminator) in the genomic DNA of the wild-type (WT) strain (negative control) and transformant BbVRF1. M, marker. (B) Semi-quantitative RT-PCR analysis of *VRF1* in the cDNA of WT and BbVRF1. The *actin* gene was used as an internal reference. (C) Western blot of the secretory venom protein VRF1 in Sabouraud dextrose broth (SDB) of WT and BbVRF1. M, marker. (D to F) qRT-PCR analysis of several AMP genes regulated by the Toll pathway in IOZCAS-Ha-I cells (*H. armigera* cell line) 72 h after coculture with B. bassiana strains. Mock, cells infected with 1 × PBS. WT, cells infected with WT (B. bassiana ARSEF 2860). BbVRF1, cells infected with transformant BbVRF1. Data are shown as means ± standard deviation (SD) of three biological replicates. Ordinary one-way analysis of variance (ANOVA) with Tukey’s multiple-comparison test was performed (*P* ≤ 0.05).

The *VRF1*-inclusive cassette (3.5 kb) was detected in the genomic DNA of the putative transformant via PCR analysis and verified by sequencing (Fig. 2A). cDNA of the fungus was synthesized by reverse transcription of total RNA, and then semi-quantitative reverse transcription-PCR (RT-PCR) confirmed that the exogenous virulence factor *VRF1* had been successfully transcribed into the transformant with *actin* (GenBank ID: XM_008599957.1) as an internal reference (Fig. 2B, Table S1). Using Western blotting, around 100 kDa VRF1 (expressed with an mCherry tag at the C terminus) was detected in the supernatant (1) (Fig. 2C). Therefore, the transcription of *VRF1* and secretion after translation by transformant BbVRF1 were confirmed.

In the screening process, we chose dozens of candidates of transformants after A. tumefaciens-mediated transformation on PDA plates with phosphinothricin (Fig. S1A), and transferred them to new PDA plates (phosphinothricin) for the second screening. After this, we checked the inserted cassette and expression levels of *VRF1* of five single colonies with the same morphology as the B. bassiana ARSEF 2860 (WT, wild-type strain) using PCR and RT-PCR (Fig. S1B and C). Strains 3 and 4 obtained higher *VRF1* expression on the transcriptional level, and strain 4 had a higher protein expression level than strain 3 (Fig. S1D). In addition, there were no significant differences in colony growth rate and spore output between strain 4 and the WT (Fig. S2). Thus, we used strain 4 as BbVRF1 in the following experiments.

Morphologies and the levels of mycelial growth and conidiation were similar between the WT and BbVRF1 (Fig. S3). We examined VRF1 expression during infection with 5th-instar *H. armigera* using BbVRF1, and VRF1 accumulated in the hemolymph at 2 and 3 d after infection. *H. armigera* was nearly dead at 4 d after BbVRF1 infection, and there were very few hemocytes in the hemocoel (Fig. S4).

### VRF1 secreted by transformant BbVRF1 enters host cells and then suppresses encapsulation and AMP expression.

As previously determined, venom protein VRF1 derived from the venom apparatus of *M. mediator* can enter host hemocytes at 6 h post-parasitism (35). We tested the effects of WT and BbVRF1 on *H. armigera*. The mRNA abundance of AMPs in *H. armigera* cells, which was induced by B. bassiana, was measured 72 h after coculture with WT or BbVRF1 by quantitative real-time PCR (qRT-PCR). The results showed that the expression of AMPs was markedly suppressed in the BbVRF1-infected groups (Fig. 2D to F).

**FIG 3:**
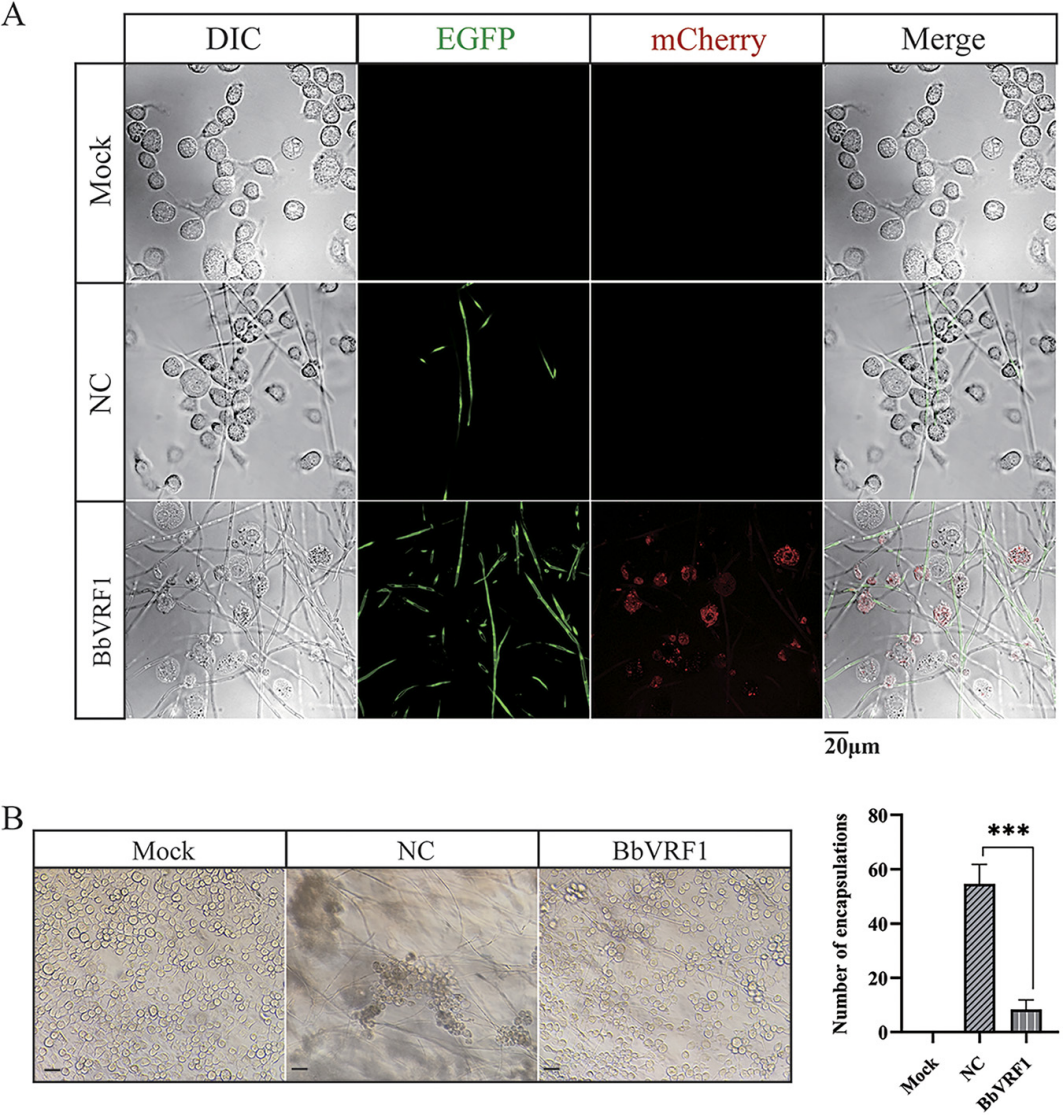
BbVRF1 secretes VRF1 into the host cells. (A) Live-cell fluorescence imaging of a coculture of IOZCAS-Ha-I cells (*H. armigera* cell line) and B. bassiana. Here, 1 × PBS and conidia of WT and BbVRF1 were added to IOZCAS-Ha-I cells, incubated for 72 h, and then observed via differential interference contrast (DIC) microscopy and excitation of EGFP and mCherry. Mock, coculture of 1 × PBS and cells. NC, negative control, coculture of Bb-EGFP (B. bassiana strain transformed with the empty pPK2-Bar-EGFP). BbVRF1, a B. bassiana strain transformant integrated with *VRF1* tailed by the fluorescent tag *mCherry*. Scale bar = 20 μm. (B) Infected IOZCAS-Ha-I cells were detected after incubation with 1 × PBS and conidia of NC and BbVRF1 for 72 h. Main views of different groups are shown and the number of encapsulations was counted. Scale bar = 50 μm. Error bars represent the means ± SD from three replicates. Differences between treatments were compared by one-way ANOVA followed by Tukey’s test for multiple comparisons (***, *P* ≤ 0.001).

To determine whether VRF1 secreted by BbVRF1 entered *H. armigera* cells, conidia of WT and BbVRF1 were cultured with IOZCAS-Ha-I (a cell line of *H. armigera*). After 72 h of coculture, the developed mycelia displayed positive green fluorescent signals in both the negative control (NC), a strain of B. bassiana ARSEF 2860 transformed with empty plasmid pPK2-Bar-EGFP, and BbVRF1, as observed using a fluorescence microscope, while VRF1, indicated by the red spots, was only detected in the host cells cultured with BbVRF1. Furthermore, the intracytoplasmic red spots indicated that VRF1 entered *H. armigera* cells (Fig. 3A).

The presence of rVRF1 was shown to significantly decrease the encapsulation rate of *M. mediator* eggs in *H. armigera* after parasitism. To further determine whether VRF1 secreted by BbVRF1 prevented B. bassiana from being encapsulated, resulting in a shorter life expectancy of infected *H. armigera*, we observed cells cultured with the NC or BbVRF1 and counted the encapsulations (Fig. 3B). As expected, in the coculture of NC and cells, cells were recruited to encapsulate mycelia, and the encapsulations were significantly greater in number and dimension than in the coculture of BbVRF1 and cells (Fig. 3B).

Hence, we concluded that VRF1 secreted by BbVRF1 functioned in the same manner as venom protein secreted by *M. mediator*, and that VRF1 could enter host cells to facilitate the infection of B. bassiana.

### Virulence of BbVRF1 against *Helicoverpa armigera*, the natural host of *Microplitis mediator*.

**FIG 4:**
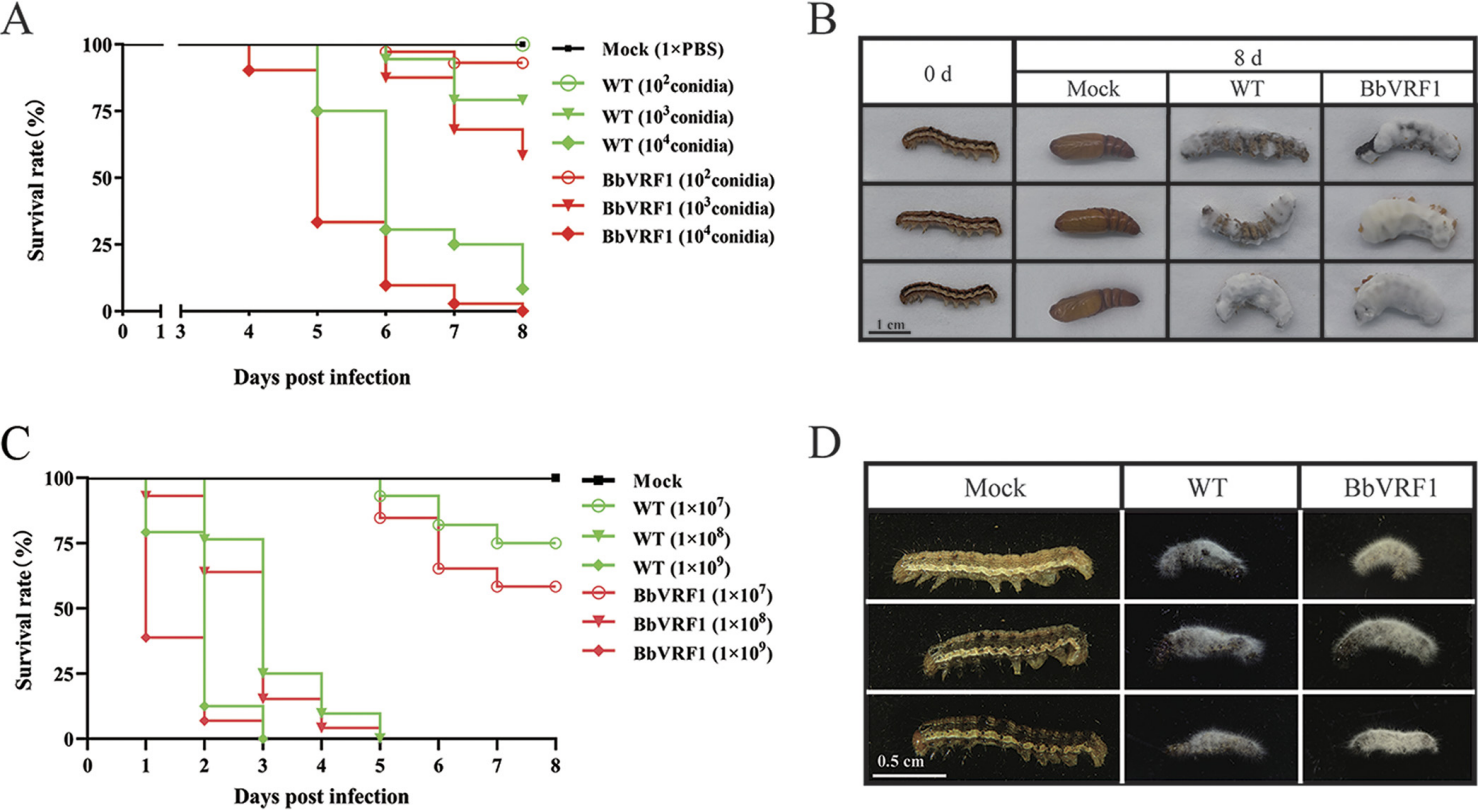
Insecticidal activity of WT and transformant BbVRF1 against *H. armigera* larvae in laboratory conditions. (A) Survival rates of *H. armigera* larvae after injection of 5 μL of WT (B. bassiana ARSEF 2860) and BbVRF1 conidia suspensions (1 × PBS) at three spore concentration gradients (2 × 10^4^, 2 × 10^5^, and 2 × 10^6^ conidia mL^−1^), for a final 1 × 10^2^, 1 × 10^3^, and 1 × 10^4^ conidia per larva (*n* = 72). (B) Symptoms of *H. armigera* 8 days after treatment. BbVRF1-treated larvae died in a shorter time compared to WT-infected larvae, and BbVRF1 hyphae were greater in number and stronger than those of the WT strain. (C) Survival rates of *H. armigera* larvae after topical infection of WT and BbVRF1 conidia suspensions (0.1% Tween 80) at three spore concentration gradients (1 × 10^7^, 1 × 10^8^, and 1 × 10^9^ conidia mL^−1^) (*n* = 72). (D) Symptoms of *H. armigera* 8 days after treatment.

The suspensions of WT and transformant BbVRF1 conidia (2 × 10^4^, 2 × 10^5^, 1 × 10^6^, 2 × 10^6^, and 5 × 10^6^ conidia mL^−1^) were injected into 5th-instar *H. armigera* larvae (5 μL per insect) under laboratory conditions to analyze their entomopathogenic activities. Compared with the WT, the transformant strain BbVRF1 displayed increased virulence. In injected inoculation, the LT_50_ was significantly shortened to 4.79 days (about 84% of WT) (injected with 1 × 10^4^ conidia), and the LD_50_ was 1.23 × 10^3^ conidia (about 2.36-fold decrease of WT) in BbVRF1 compared to the estimate for the WT strain (calculated by the bioassay data under gradient doses: 1 × 10^2^, 1 × 10^3^, 5 × 10^3^, 1 × 10^4^, and 2.5 × 10^4^ conidia) (Tables S2 to S4, Fig. 4A). The infection was timed on the first day of the 5th instar, and the control groups pupated normally after 8 d. The survival times of the BbVRF1-infected groups were shorter than that of the WT group, and most of the mycelia of BbVRF1 breached the body wall and grew faster and stronger than those of the WT group (Fig. 4B), which might have been inhibited by encapsulations and host AMPs during the infection process.

To determine the performance of BbVRF1 via the natural route, we used conidia suspensions to carry out topical infection by gradient concentration (1 × 10^7^, 1 × 10^8^, and 1 × 10^9^ conidia mL^−1^) on 3rd-instar *H. armigera* larvae. Survival curves are shown in Fig. 4C. The survivorship curve showed that VRF1 also increased the virulence of B. bassiana in natural infection. Like in infection by injection, *H. armigera* topically infected with BbVRF1 also developed stronger mycelia (Fig. 4D).

**FIG 5:**
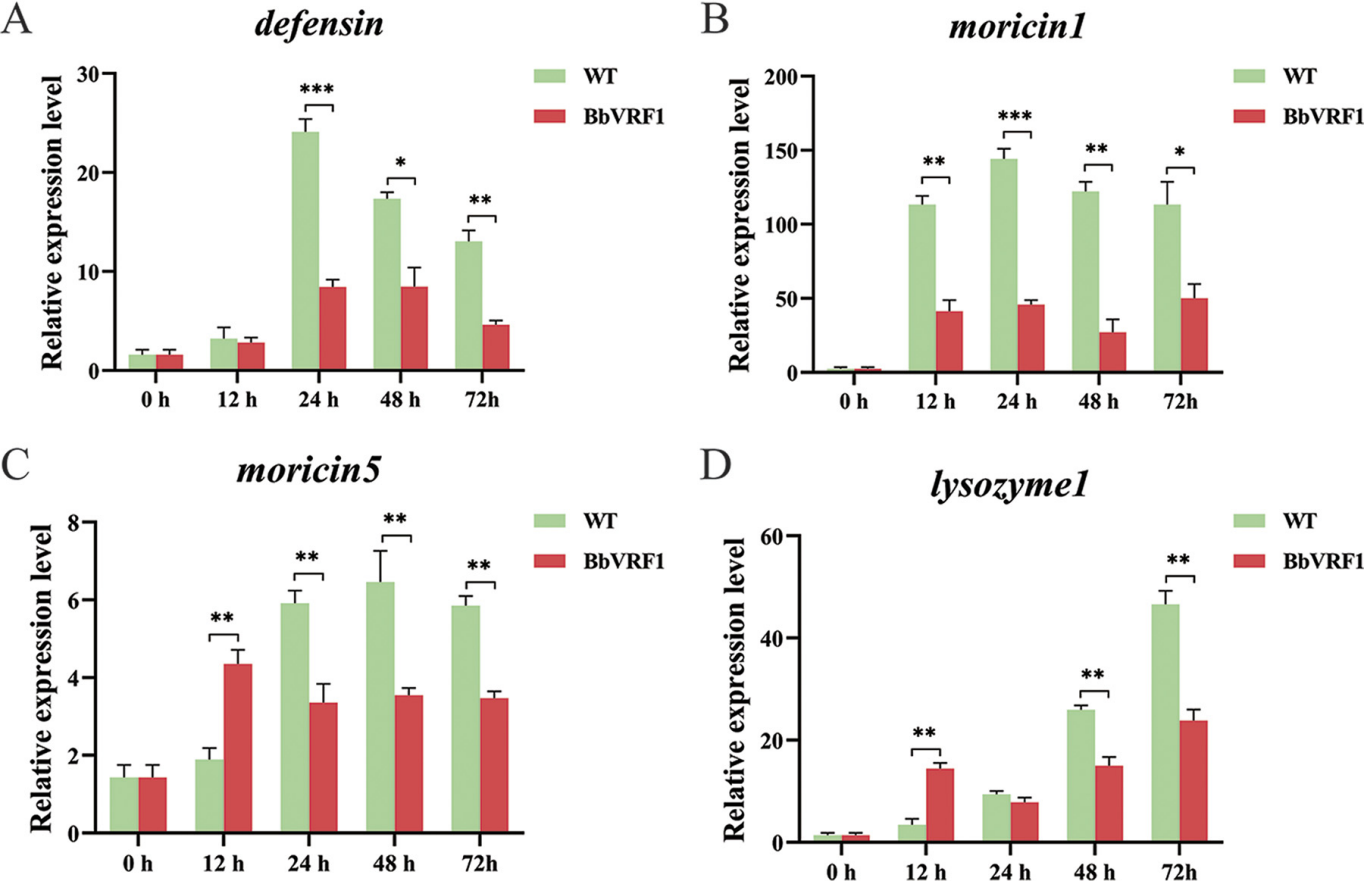
Expression levels of AMPs in hemocytes after infection. (A to C) Time-course analysis of several AMP genes regulated by the Toll pathway in *H. armigera* hemocytes after infection by qRT-PCR. WT, 5th-instar *H. armigera* larvae injected with 10^4^ conidia of WT (B. bassiana ARSEF 2860). BbVRF1, 5th-instar *H. armigera* larvae injected with 10^4^ conidia of BbVRF1. Data are shown as means ± standard error of the mean (SEM) of three biological replicates (*n* = 24). Asterisks indicate significant differences: *, *P* ≤ 0.05; **, *P* ≤ 0.01; ***, *P* ≤ 0.001 (Student’s *t* test).

To verify whether VRF1 secreted by B. bassiana was effective *in vivo*, we tested the relative expression levels of AMP genes in the hemocytes of *H. armigera* infected with WT and BbVRF1 (Fig. 5). As shown in the time course results, as in the *in vitro* experiment in the cell line, *moricin-1*, *moricin-5*, and *lysozyme-1* were significantly downregulated in the hemocytes of BbVRF1-infected *H. armigera* compared to the control group infected with WT.

### Virulence of BbVRF1 against *Spodoptera frugiperda*, an invasive species.

In a previous experiment, we found that nearly 70% of 2nd-instar *S. frugiperda* larvae (an unsuitable/unnatural host) rapidly died of parasitism by *M. mediator* in 12 h (Fig. S5). Because PDV and teratocytes take time to affect hosts, this death in such a short time was probably due to the venom of *M. mediator*. This led us to consider that the venom of *M. mediator* might be more deadly to *S. frugiperda* than to *H. armigera* and could be a powerful weapon to kill *S. frugiperda*.

**FIG 6:**
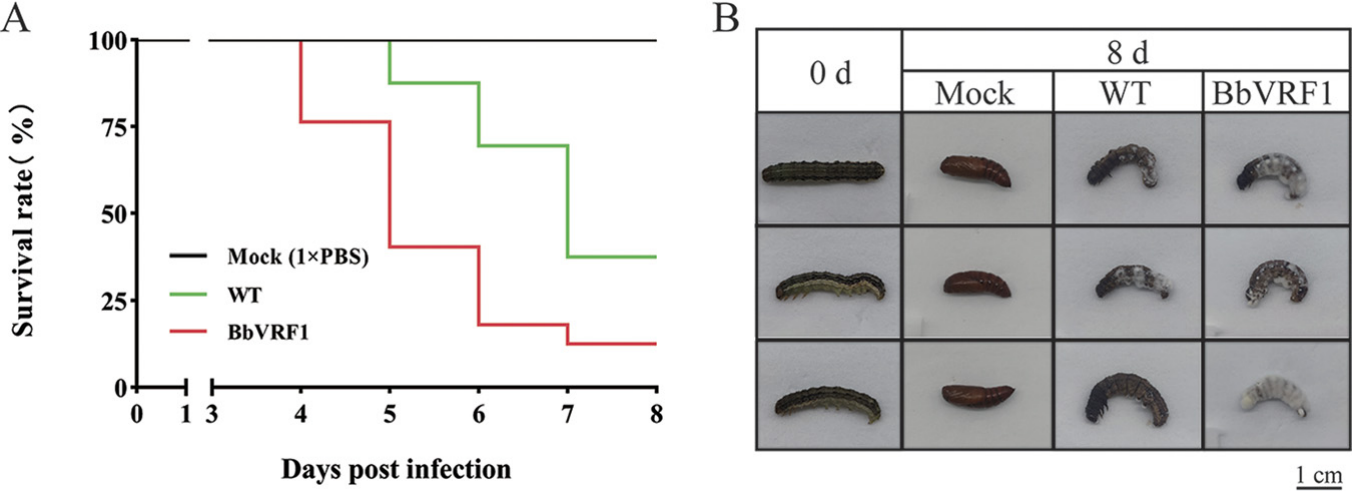
*M. mediator* is more lethal against the unnatural host *S. frugiperda* than against the natural host *H. armigera*. (A) Survival rates of *S. frugiperda* larvae after injection of 5 μL of wild-type (WT) and BbVRF1 conidia suspensions (2 × 10^6^ conidia mL^−1^) for a final 10^4^ conidia per larva (*n* = 72). (B) Symptoms of *S. frugiperda* 8 days after treatment.

To verify whether virulence against in *S. frugiperda* could be enhanced by the venom protein VRF1, we carried out bioassays of WT (B. bassiana ARSEF 2860) and BbVRF1 against *S. frugiperda* in the same manner as for *H. armigera*. As the survival curve illustrates, BbVRF1 was more toxic than the WT (Fig. 6A). Compared with the WT, the LT_50_ of BbVRF1 was shortened to 4.81 days (about 73% of the WT) (injected with 1 × 10^4^ conidia) and the LD_50_ was decreased to 1.69 × 10^3^ conidia (about 3.45-fold decrease compared to the WT) (calculated by the bioassay data under gradient doses: 1 × 10^2^, 1 × 10^3^, 5 × 10^3^, 1 × 10^4^, and 2.5 × 10^4^ conidia) (Table S3-S5). As shown in Fig. 6B, BbVRF1-infected *S. frugiperda* died early, with more and stronger B. bassiana mycelia. VRF1 seems to be more efficient for increasing the virulence of B. bassiana against S. *frugiperda* than that against *H. armigera*.

## DISCUSSION

Based on previous studies, a better understanding of the compositions and functions of *M. mediator* venom could contribute to fungal virulence by introducing the venom factors into the fungi (34, 35, 44). As shown above, we show that the engineered entomopathogenic fungi B. bassiana (BbVRF1) secreted a venom protein VRF1 that entered host insect cells, where it attenuated host immunity and accelerated fungal infection. This was an effort to equip a fungus with the arsenal of parasitoid wasps. Constitutive expression of VRF1 by BbVRF1 in *H. armigera* (the natural host of *M. mediator*, the origin of VRF1) resulted in a 2.36-fold decrease in the LD_50_ and 84% of the original LT_50_. Surprisingly, VRF1 functioned more effectively in controlling *S. frugiperda* (an invasive species), resulting in a 3.45-fold decrease in the LD_50_ and 73% of the original LT_50_. The results of this study indicated that parasitoid wasps could be another potential resource to screen valuable factors for increasing the efficacy of entomopathogenic fungi (1, 7–15).

Specific engineering of entomopathogenic fungi with targeted toxins often produces promising results. Many researchers have attempted to use neurotoxins to enhance fungal virulence and eventually succeeded, such as in the following examples: integrated AaIT increased the virulence of M. anisopliae, resulting in a 22-fold decrease in the LC_50_ and a 28% reduction in the LT_50_ for Manduca sexta, and an 8.9-fold decrease in the LC_50_ and a 38% reduction in the LT_50_ for Aedes aegypti; integrated Bmkit increased the virulence of Lecanicillium lecanii, resulting in a 7.1-fold decrease in the LC_50_ and a 26.5% reduction in the LT_50_ for Aphis gossypii; integrated LqhIT2 increased the virulence of Metarhizium acridum, resulting in a 22.6-fold decrease in the LC_50_ and 29.6% reduction in the LT_50_ for Locusta migratoria manilensis (5, 45, 46). Furthermore, the ability of fungi to penetrate the cuticle of pests is also a route for increasing virulence, such as in Pr1, which increased the virulence of M. anisopliae with no significant difference in the LC_50_ but a 25% reduction in the LT_50_ for *M. sexta*; and Bbchit1, which increased the virulence of *B.* bassiana and resulted in a 5.45- to 8.97-fold decrease in the LC_50_ and a 20.2% reduction in the LT_50_ for Myzus persicae (2, 4). In addition, proteins that disturb insect immune systems, such as VRF1, are ideal for increasing virulence, including the following factors in *B.* bassiana: Spn43Ac, which resulted in an approximately 3-fold decrease in the LD_50_ and 24% reduction in the LT_50_ for Galleria mellonella; TMOF (trypsin-modulating oostatic factor), which resulted in a 1.6-fold decrease in the LD_50_ and a 25% reduction in the LT_50_ for Anopheles gambiae; and BiVSP, which resulted in an 11.3-fold decrease in the LC_50_ and a 55% reduction in the LT_50_ for Spodopetra exigua (7–9). Thus, we speculate that the rich variety of resources in nature for screening virulence factors could provide the foundation for building a scientific system of engineering fungi to overcome pest resistance. Considering their environmental benefits, fungal biopesticides are cost-effective and suitable for large-scale production (1).

*M. mediator*, a type of endoparasitoid wasp that uses venom to control its hosts for the development of its offspring, usually parasitizes once per host in nature; however, excessive parasitization, in which one host is parasitized by two or more wasps, always exists in the laboratory setting because of the relatively higher rearing density, causing the death of the host within 12 h. We assume that there is a threshold dose of venom that can cause host death. This means that venom proteins which disturb host immunity at low concentrations and poison the host at high concentrations are ideal partners for entomopathogenic fungi to create engineered fungi which persistently express integrated toxic proteins. Surprisingly, the bioassays showed that lethality against the alien host *S. frugiperda* was higher than that against the natural host *H. armigera*, whether parasitized by *M. mediator* or infected by B. bassiana engineered with *M. mediator* VRF1 (Fig. 4 and 6). Based on this, we hypothesize that the natural host was better adapted to the venom of its parasitoid wasp due to long-term coevolution; this suggests that, as virulence factors, venom proteins are more effective against pest insects outside the host range of their original parasitoid wasp (47).

Genomic analyses of entomopathogenic fungi, pest insects, pest insect predators, and parasitoid wasps offer the opportunity for more efficient fungal genetic engineering. We can design engineered strains for different applications by choosing diverse modules of basic strains with narrow or wide host ranges; for example, arsenals including chitinases, neurotoxins, and immune suppression factors; and fungal defenses for tolerance to abiotic stresses such as UV radiation and heat. Research on the genomes of parasitoid wasps is relatively rare, which may be partly because most species are rather small and have a complex life cycle. As technologies develop and sample volume requirements become lower, increasing data on parasitoid wasps are being published, providing another library of virulence factors for fungal engineering.

In summary, instead of determining that the venom proteins of parasitoid wasps facilitate fungal infection in their hosts when injected, in this study, we first engineered a fungus integrated with venom protein derived from a parasitoid wasp and then demonstrated that it was more virulent than the wild type (35, 48). With more knowledge of the functions of venom protein, parasitoid wasps could become an inexhaustible arsenal for genetic modification, increasing the economic value and broadening the application of fungal pesticides.

## MATERIALS AND METHODS

### Insect rearing and cell line culture.

*M. mediator*, *H. armigera*, and *S. frugiperda* were all obtained from laboratory populations and reared in a constant-climate chamber under standard conditions of 26 ± 1°C, 60% relative humidity, and a 14/10-h light/dark cycle. Larval *H. armigera* and *S. frugiperda* were fed on an artificial diet. All adult insects were fed on 10% hydromel.

The IOZCAS-Ha-I (an *H. armigera* cell line) was maintained in Sf-900 II serum-free medium (Thermo Fisher Scientific, USA) with 10% fetal bovine serum (Sigma-Aldrich, USA).

### Microbial cultures and growth media.

The WT and transformants of B. bassiana ARSEF 2860 were maintained on PDA (Coolaber, China) at 26°C. For liquid culture, fungi were inoculated into Sabouraud dextrose broth (SDB; Solarbio, China) and incubated in a rotatory shaker at 26°C. For A. tumefaciens-mediated transformation, the fungi were cultured with A. tumefaciens AGL1 on solid induction medium (KH_2_PO_4_, 1.45 g L^−1^; K_2_HPO_4_, 2.05 g L^−1^; NaCl, 0.15 g L^−1^; MgSO_4_·7H_2_O, 0.5 g L^−1^; CaCl_2_, 0.05 g L^−1^; FeSO_4_·7H_2_O, 0.0025 g L^−1^; (NH_4_)_2_SO_4_, 0.5 g L^−1^; glucose, 1.8 g L^−1^; glycerol, 5 mL/L; 2-(N-morpholino)ethanesulfonic acid 40 mM; acetosyringone, 200 μM). For the proteolytic assay, 1/4 SDAY medium (Hopebio, China) and Czapek-Dox medium (Coolaber, China) were used.

The strain A. tumefaciens AGL1 was used for fungal transformation. LB medium was used for A. tumefaciens AGL1 screening, and liquid induction medium (glucose concentration was half of the solid induction medium) was used for A. tumefaciens AGL1 culture before coculture with *B.* bassiana.

### Vector construction and fungal transformation.

The origin of vector pPK2-Bar-EGFP was pPK2 (49), which was used for A. tumefaciens-mediated transformation. The reconstructed vector pPK2-Bar-EGFP included new elements: phosphinothricin acetyltransferase (*bar*) for screening; enhanced green fluorescent protein (*egfp*) for fluorescence of transformants; and the *gpdA* promoter and *trpC* terminator for transcriptional expression of the inserted gene.

*VRF1* was cloned as described previously (35). For secreted expression, the signal peptide of *VRF1* was replaced with *Bbsp* (signal peptide of chitinase of B. bassiana; GenBank ID: XM_008601414.1). Segments (linearized pPK2-Bar-EGFP, *Bbsp*, *VRF1*, and *mCherry*) were amplified with special primers with adaptors via Hi-Fi PCR, then fused using a ClonExpress MultiS one-step cloning kit (Vazyme, China).

The exogenous gene expression cassette located on pPK2-Bar-EGFP-VRF1 was transformed into the genome of B. bassiana using a protocol from a previous study (50, 51), and transformants were selected by phosphinothricin (60 μg/mL) and EGFP fluorescence.

### Characterization of *B. bassiana* transformant.

The cassette with *VRF1* in the transformants was examined by extracting genomic DNA from the mycelia, using PCR with the primers VRF1-F/R (Table S1). cDNA of BbVRF1 was synthesized by reverse transcription of total RNA extracted by TRIzol and used to examine *VRF1* expression in BbVRF1 with the primers RT-VRF1-F/R and RT-actin-F/R for the internal reference by RT-PCR (Table S1).

To investigate whether there were differences in virulence-related phenotypes between the WT and transformants, we measured colony growth rates and spore output. After the conidia suspensions (1 × 10^6^ conidia mL^−1^) had been prepared, 1 μL of suspension was pipetted onto the centers of PDA plates. It was then inverted, and the plates were placed into an incubator set at 26°C. There were five repeats of each strain. Colony diameters of the plates were measured every 1 day until the 20th day. Next, we prepared suspensions of the plates separately with equal amounts of sterile water. Spore outputs were calculated according to the concentrations of the suspensions using a blood counting chamber.

For Western blot, WT and BbVRF1 strains were incubated in SDB at 26°C and 200 rpm for 72 h. The fungal cultures were filtered by 3M filter papers and syringe filters (0.45 μm), then incubated with anti-mCherry Nanobody agarose beads (KTlife, China) for 2 h. Next, the agarose beads were heated and denatured in Omni-Easy protein sample loading buffer (Epizyme, China) at 95°C for 5 min, and then centrifuged at 12,000 × *g* for 1 min. The supernatants were subjected to 10% discontinuous sodium dodecyl sulfate polyacrylamide gel electrophoresis (SDS-PAGE) and the separated proteins were electroblotted onto a polyvinylidene difluoride (PVDF) membrane (Invitrogen, USA). Proteins were identified using anti-VRF1 monoclonal antibody and chemiluminescence (SuperSignal West Pico PLUS; Thermo Fisher Scientific, USA) as previously described (35). Gels were calibrated using a Multicolor pre-stained protein ladder (Epizyme, China).

### Fluorescence localization of VRF1 secreted by BbVRF1.

After scraping the fungal colonies on the surface of PDA plates, the mixtures of mycelia and spores were shaken with sterile water and then filtered with sterile 3M filter paper to harvest conidia of the WT and BbVRF1 strains. *H. armigera* cells (IOZCAS-Ha-I) were cultured in μ-slide 8-well chambers (ibidi GmbH, Germany) by adherent culture (6,000 cells per well). Conidia of WT and BbVRF1 strains were inoculated into the cells (100 conidia per well) and incubated for 72 h. Live cell images were captured by an Andor Dragonfly (Andor, United Kingdom).

### Fungi encapsulation assay.

*H. armigera* cells were cultured in six-well culture plates (60,000 cells per well) with WT and BbVRF1 conidia (1,000 conidia per well) for 72 h. The number of encapsulated mycelia was counted under a light microscope. The encapsulation rate was analyzed using one-way analysis of variance followed by Tukey’s multiple-comparison test.

### qRT-PCR analysis of AMPs.

Conidia of WT and BbVRF1 strains were harvested, and the concentrations of their suspensions were adjusted to 2 × 10^6^ conidia mL^−1^. For the *in vitro* experiment, *H. armigera* cells were cultured in six-well culture plates (60,000 cells per well) and infected with 1 μL suspension of WT or BbVRF1. The mock-infected (infected with 1 × phosphate-buffered saline [PBS]), WT-infected (infected with WT strain), and BbVRF1-infected (infected with BbVRF1 strain) cells were collected 72 h later. For the *in vivo* experiment, 5th-instar *H. armigera* larvae were injected with 5 μL conidia suspension (2 × 10^6^ conidia mL^−1^) of WT and BbVRF1. The hemocytes were collected at 0 h, 12 h, 24 h, 48 h and 72 h after infection. RNA was extracted from samples using the RNA Easy Fast tissue/cell kit (Tiangen, China). The RNA was treated using the HiScript III All-in-One RT SuperMix Perfect for RT-qPCR (Vazyme, China) for reverse transcription, and qRT-PCR was performed using the StepOne Plus RT-qPCR system (Applied Biosystems, USA) and *Taq* Pro Universal SYBR qPCR master mix (Vazyme, China) according to the manufacturers’ manuals, using the primers listed in Table S1. The *rps3* gene was used as an internal reference (35).

### Wasp parasitism.

Second-instar larvae of *H. armigera* and *S. frugiperda* were selected for parasitism. Each treatment used 100 larvae. Every larva was placed into an overturned 50-mL centrifuge tube containing 50 female *M. mediator* and then removed upon observation of parasitization. All hosts were reared individually in 24-well plates to grow further until one of three phenotypes was observed: success, death by parasitism, or failure.

### Bioassay.

To verify the virulence enhancement of BbVRF1, insect bioassays were performed using newly emerged last-instar larvae of *H. armigera* and *S. frugiperda*. Conidia suspensions (2 × 10^4^, 2 × 10^5^, 1 × 10^6^, 2 × 10^6^, and 5 × 10^6^ conidia mL^−1^) of WT and BbVRF1 strains were prepared for injection assays. Before injection, every larva was sterilized with 75% alcohol wipes and anesthetized by placing it on ice. Injections were made using a microsyringe. Each treatment had three replicates with 24 larvae, and the bioassays were repeated three times. Every larva was injected with 5 μL of conidia suspensions (final doses: 1 × 10^2^, 1 × 10^3^, 5 × 10^3^, 1 × 10^4^, and 2.5 × 10^4^ conidia) at the base of the third pair of prolegs. Insect mortality was recorded every 24 h after injection. For topical infection, bioassays were performed using newly emerged 3rd-instar *H. armigera* larvae. Conidia suspensions (1 × 10^7^, 1 × 10^8^, and 1 × 10^9^ conidia mL^−1^) of WT and BbVRF1 strains were prepared for topical infection with 0.1% Tween 80. Each larva was soaked in a conidia suspension for 15 s, then clipped out without excess suspension by wiping. The conidia suspension was vortexed every 2 min to prevent the conidia from sinking to the bottom. Differences between the WT and BbVRF1 for *H. armigera* and *S. frugiperda* were estimated by a log-rank test. LD_50_ and LT_50_ were determined by probit analysis with SPSS (version 21).

## References

[1] Lovett B, St Leger RJ. 2018. Genetically engineering better fungal biopesticides. Pest Manag Sci 74:781–789. doi:10.1002/ps.4734. 28905488

[2] Fang W, Leng B, Xiao Y, Jin K, Ma J, Fan Y, Feng J, Yang X, Zhang Y, Pei Y. 2005. Cloning of *Beauveria bassiana* chitinase gene Bbchit1 and its application to improve fungal strain virulence. Appl Environ Microbiol 71:363–370. doi:10.1128/AEM.71.1.363-370.2005. 15640210 PMC544255

[3] Fang W, Feng J, Fan Y, Zhang Y, Bidochka MJ, Leger RJ, Pei Y. 2009. Expressing a fusion protein with protease and chitinase activities increases the virulence of the insect pathogen *Beauveria bassiana*. J Invertebr Pathol 102:155–159. doi:10.1016/j.jip.2009.07.013. 19666027

[4] St Leger RJ, Joshi L, Bidochka MJ, Roberts DW. 1996. Construction of an improved mycoinsecticide overexpressing a toxic protease. Proc Natl Acad Sci USA 93:6349–6354. doi:10.1073/pnas.93.13.6349. 8692818 PMC39025

[5] Wang C, St Leger RJ. 2007. A scorpion neurotoxin increases the potency of a fungal insecticide. Nat Biotechnol 25:1455–1456. doi:10.1038/nbt1357. 17994009

[6] Liu YJ, Liu J, Ying SH, Liu SS, Feng MG. 2013. A fungal insecticide engineered for fast per os killing of caterpillars has high field efficacy and safety in full-season control of cabbage insect pests. Appl Environ Microbiol 79:6452–6458. doi:10.1128/AEM.01594-13. 23956386 PMC3811226

[7] Kim JS, Choi JY, Lee JH, Park JB, Fu Z, Liu Q, Tao X, Jin BR, Skinner M, Parker BL, Je YH. 2013. Bumblebee venom serine protease increases fungal insecticidal virulence by inducing insect melanization. PLoS One 8:e62555. doi:10.1371/journal.pone.0062555. 23626832 PMC3633896

[8] Yang L, Keyhani NO, Tang G, Tian C, Lu R, Wang X, Pei Y, Fan Y, Brakhage AA. 2014. Expression of a Toll signaling regulator serpin in a mycoinsecticide for increased virulence. Appl Environ Microbiol 80:4531–4539. doi:10.1128/AEM.01197-14. 24837378 PMC4148788

[9] Kamareddine L, Fan Y, Osta MA, Keyhani NO. 2013. Expression of trypsin modulating oostatic factor (TMOF) in an entomopathogenic fungus increases its virulence towards *Anopheles gambiae* and reduces fecundity in the target mosquito. Parasit Vectors 6:1–5. doi:10.1186/1756-3305-6-22. 23336669 PMC3571938

[10] Lovett B, St Leger RJ. 2015. Stress is the rule rather than the exception for *Metarhizium*. Curr Genet 61:253–261. doi:10.1007/s00294-014-0447-9. 25239135

[11] Ortiz-Urquiza A, Keyhani NO. 2015. Stress response signaling and virulence: insights from entomopathogenic fungi. Curr Genet 61:251–251. doi:10.1007/s00294-015-0485-y. 25836595

[12] Liao X, Lu HL, Fang W, St Leger RJ. 2014. Overexpression of a *Metarhizium robertsii* HSP25 gene increases thermotolerance and survival in soil. Appl Microbiol Biotechnol 98:777–783. doi:10.1007/s00253-013-5360-5. 24265026

[13] Tseng MN, Chung PC, Tzean SS. 2011. Enhancing the stress tolerance and virulence of an entomopathogen by metabolic engineering of dihydroxynaphthalene melanin biosynthesis genes. Appl Environ Microbiol 77:4508–4519. doi:10.1128/AEM.02033-10. 21571888 PMC3127726

[14] Shang Y, Duan Z, Huang W, Gao Q, Wang C. 2012. Improving UV resistance and virulence of *Beauveria bassiana* by genetic engineering with an exogenous tyrosinase gene. J Invertebr Pathol 109:105–109. doi:10.1016/j.jip.2011.10.004. 22024554

[15] Zimmermann G. 2007. Review on safety of the entomopathogenic fungi *Beauveria bassiana* and *Beauveria brongniartii*. Biocontrol Sci Technol 17:553–596. doi:10.1080/09583150701309006.

[16] Ortiz-Urquiza A, Keyhani NO. 2016. Molecular genetics of *Beauveria bassiana* infection of insects. Adv Genet 94:165–249. doi:10.1016/bs.adgen.2015.11.003. 27131326

[17] Zhang Y, Zhang J, Jiang X, Wang G, Luo Z, Fan Y, Wu Z, Pei Y. 2010. Requirement of a mitogen-activated protein kinase for appressorium formation and penetration of insect cuticle by the entomopathogenic fungus *Beauveria bassiana*. Appl Environ Microbiol 76:2262–2270. doi:10.1128/AEM.02246-09. 20139313 PMC2849248

[18] Cito A, Barzanti GP, Strangi A, Francardi V, Zanfini A, Dreassi E. 2016. Cuticle-degrading proteases and toxins as virulence markers of *Beauveria bassiana* (Balsamo) Vuillemin. J Basic Microbiol 56:941–948. doi:10.1002/jobm.201600022. 27198125

[19] Wanchoo A, Lewis MW, Keyhani NO. 2009. Lectin mapping reveals stage-specific display of surface carbohydrates in in vitro and haemolymph-derived cells of the entomopathogenic fungus *Beauveria bassiana*. Microbiology (Reading) 155:3121–3133. doi:10.1099/mic.0.029157-0. 19608611

[20] Lemaitre B, Hoffmann J. 2007. The host defense of *Drosophila melanogaster*. Annu Rev Immunol 25:697–743. doi:10.1146/annurev.immunol.25.022106.141615. 17201680

[21] Valanne S, Wang JH, Ramet M. 2011. The *Drosophila* Toll signaling pathway. J Immunol 186:649–656. doi:10.4049/jimmunol.1002302. 21209287

[22] Xiong GH, Xing LS, Lin Z, Saha TT, Wang C, Jiang H, Zou Z. 2015. High throughput profiling of the cotton bollworm *Helicoverpa armigera* immunotranscriptome during the fungal and bacterial infections. BMC Genomics 16:321. doi:10.1186/s12864-015-1509-1. 26001831 PMC4490664

[23] Yi HY, Chowdhury M, Huang YD, Yu XQ. 2014. Insect antimicrobial peptides and their applications. Appl Microbiol Biotechnol 98:5807–5822. doi:10.1007/s00253-014-5792-6. 24811407 PMC4083081

[24] Beckage NE, Gelman DB. 2004. Wasp parasitoid disruption of host development: implications for new biologically based strategies for insect control. Annu Rev Entomol 49:299–330. doi:10.1146/annurev.ento.49.061802.123324. 14651466

[25] Burke GR, Strand MR. 2014. Systematic analysis of a wasp parasitism arsenal. Mol Ecol 23:890–901. doi:10.1111/mec.12648. 24383716 PMC4120856

[26] Colinet D, Mathe-Hubert H, Allemand R, Gatti JL, Poirie M. 2013. Variability of venom components in immune suppressive parasitoid wasps: from a phylogenetic to a population approach. J Insect Physiol 59:205–212. doi:10.1016/j.jinsphys.2012.10.013. 23103980

[27] Moreau SJ, Asgari S. 2015. Venom proteins from parasitoid wasps and their biological functions. Toxins (Basel) 7:2385–2412. doi:10.3390/toxins7072385. 26131769 PMC4516919

[28] Strand MR, Burke GR. 2020. Polydnaviruses: evolution and function. Curr Issues Mol Biol 34:163–182. doi:10.21775/cimb.034.163. 31167960

[29] Strand MR. 2014. Teratocytes and their functions in parasitoids. Curr Opin Insect Sci 6:68–73. doi:10.1016/j.cois.2014.09.005. 32846683

[30] Wang ZZ, Ye XQ, Shi M, Li F, Wang ZH, Zhou YN, Gu QJ, Wu XT, Yin CL, Guo DH, Hu RM, Hu NN, Chen T, Zheng BY, Zou JN, Zhan LQ, Wei SJ, Wang YP, Huang JH, Fang XD, Strand MR, Chen XX. 2018. Parasitic insect-derived miRNAs modulate host development. Nat Commun 9:2205. doi:10.1038/s41467-018-04504-1. 29880839 PMC5992160

[31] Brown JJ, Kiuchi M, Kainoh Y, Takeda S. 1993. *In vitro* release of ecdysteroids by an endoparasitoid, *Ascogaster reticulatus* Watanabe. J Insect Physiol 39:229–234. doi:10.1016/0022-1910(93)90093-7.

[32] Shi J, Jin H, Wang F, Stanley DW, Wang H, Fang Q, Ye G. 2022. The larval saliva of an endoparasitic wasp, *Pteromalus puparum*, suppresses host immunity. J Insect Physiol 141:104425. doi:10.1016/j.jinsphys.2022.104425. 35878702

[33] Führer E, Willers D. 1986. The anal secretion of the endoparasitic larva Pimpla turionellae: sites of production and effects. J Insect Physiol 32:361–367. doi:10.1016/0022-1910(86)90049-1.

[34] Lin Z, Wang RJ, Cheng Y, Du J, Volovych O, Han LB, Li JC, Hu Y, Lu ZY, Lu Z, Zou Z. 2019. Insights into the venom protein components of *Microplitis mediator*, an endoparasitoid wasp. Insect Biochem Mol Biol 105:33–42. doi:10.1016/j.ibmb.2018.12.013. 30602123

[35] Lin Z, Cheng Y, Wang R-J, Du J, Volovych O, Li J-C, Hu Y, Lu Z-Y, Lu Z, Zou Z. 2018. A metalloprotease homolog venom protein from a parasitoid wasp suppresses the Toll pathway in host hemocytes. Front Immunol 9:2301. doi:10.3389/fimmu.2018.02301. 30405599 PMC6206080

[36] Parkinson N, Conyers C, Smith L. 2002. A venom protein from the endoparasitoid wasp *Pimpla hypochondriaca* is similar to snake venom reprolysin-type metalloproteases. J Invertebr Pathol 79:129–131. doi:10.1016/s0022-2011(02)00033-2. 12095244

[37] de Graaf DC, Aerts M, Brunain M, Desjardins CA, Jacobs FJ, Werren JH, Devreese B. 2010. Insights into the venom composition of the ectoparasitoid wasp *Nasonia vitripennis* from bioinformatic and proteomic studies. Insect Mol Biol 19:11–26. doi:10.1111/j.1365-2583.2009.00914.x. 20167014 PMC3544295

[38] Vincent B, Kaeslin M, Roth T, Heller M, Poulain J, Cousserans F, Schaller J, Poirié M, Lanzrein B, Drezen J-M, Moreau SJM. 2010. The venom composition of the parasitic wasp *Chelonus inanitus* resolved by combined expressed sequence tags analysis and proteomic approach. BMC Genomics 11:693. doi:10.1186/1471-2164-11-693. 21138570 PMC3091792

[39] Colinet D, Deleury E, Anselme C, Cazes D, Poulain J, Azema-Dossat C, Belghazi M, Gatti JL, Poirie M. 2013. Extensive inter- and intraspecific venom variation in closely related parasites targeting the same host: the case of *Leptopilina* parasitoids of *Drosophila*. Insect Biochem Mol Biol 43:601–611. doi:10.1016/j.ibmb.2013.03.010. 23557852

[40] Dorémus T, Urbach S, Jouan V, Cousserans F, Ravallec M, Demettre E, Wajnberg E, Poulain J, Azéma-Dossat C, Darboux I, Escoubas J-M, Colinet D, Gatti J-L, Poirié M, Volkoff A-N. 2014. Corrigendum to “Venom gland extract is not required for successful parasitism in the polydnavirus-associated endoparasitoid *Hyposoter didymator* (Hym. Ichneumonidae) despite the presence of numerous novel and conserved venom proteins” (Insect Biochem. Mol. Biol. 43 [2013], 292–307). Insect Biochem Mol Biol 44:54–54. doi:10.1016/j.ibmb.2013.07.001. 23298679

[41] Doremus T, Urbach S, Jouan V, Cousserans F, Ravallec M, Demettre E, Wajnberg E, Poulain J, Azema-Dossat C, Darboux I, Escoubas JM, Colinet D, Gatti JL, Poirie M, Volkoff AN. 2013. Venom gland extract is not required for successful parasitism in the polydnavirus-associated endoparasitoid *Hyposoter didymator* (Hym. Ichneumonidae) despite the presence of numerous novel and conserved venom proteins. Insect Biochem Mol Biol 43:292–307. doi:10.1016/j.ibmb.2012.12.010. 23298679

[42] Price DRG, Bell HA, Hinchliffe G, Fitches E, Weaver R, Gatehouse JA. 2009. A venom metalloproteinase from the parasitic wasp *Eulophus pennicornis* is toxic towards its host, tomato moth (*Lacanobia oleracae*). Insect Mol Biol 18:195–202. doi:10.1111/j.1365-2583.2009.00864.x. 19320760

[43] Crawford AM, Brauning R, Smolenski G, Ferguson CM, Barton DM, Wheeler TT, Mcculloch A. 2008. The constituents of *Microctonus* sp. parasitoid venoms. Insect Mol Biol 17:313–324. doi:10.1111/j.1365-2583.2008.00802.x. 18477245

[44] Du J, Lin Z, Volovych O, Lu Z, Zou Z. 2020. A RhoGAP venom protein from *Microplitis mediator* suppresses the cellular response of its host *Helicoverpa armigera*. Dev Comp Immunol 108:103675. doi:10.1016/j.dci.2020.103675. 32173445

[45] Ming X, Yan JZ, Xiao MZ, Jin JZ, De Liang P, Gang W. 2015. Expression of a scorpion toxin gene *BmKit* enhances the virulence of *Lecanicillium lecanii* against aphids. J Pest Sci 88:637–644. doi:10.1007/s10340-015-0644-4.

[46] Peng G, Xia Y. 2014. Expression of scorpion toxin LqhIT2 increases the virulence of *Metarhizium acridum* towards *Locusta migratoria manilensis*. J Ind Microbiol Biotechnol 41:1659–1666. doi:10.1007/s10295-014-1497-1. 25168679

[47] Asgari S, Rivers DB. 2011. Venom proteins from endoparasitoid wasps and their role in host-parasite interactions. Annu Rev Entomol 56:313–335. doi:10.1146/annurev-ento-120709-144849. 20822448

[48] Richards EH, Bradish H, Dani MP, Pietravalle S, Lawson A. 2011. Recombinant immunosuppressive protein from *Pimpla hypochondrica* venom (rVPr1) increases the susceptibility of *Mamestra brassicae* larvae to the fungal biological control agent, *Beauveria bassiana*. Arch Insect Biochem Physiol 78:119–131. doi:10.1002/arch.20447. 21948634

[49] Covert SF, Kapoor P, Lee M-h, Briley A, Nairn CJ. 2001. *Agrobacterium tumefaciens*-mediated transformation of *Fusarium circinatum*. Mycol Res 105:259–264. doi:10.1017/S0953756201003872.

[50] dos Reis MC, Pelegrinelli Fungaro MH, Delgado Duarte RT, Furlaneto L, Furlaneto MC. 2004. *Agrobacterium tumefaciens*-mediated genetic transformation of the entomopathogenic fungus *Beauveria bassiana*. J Microbiol Methods 58:197–202. doi:10.1016/j.mimet.2004.03.012. 15234517

[51] Lu D, Pava-Ripoll M, Li Z, Wang C. 2008. Insecticidal evaluation of *Beauveria bassiana* engineered to express a scorpion neurotoxin and a cuticle degrading protease. Appl Microbiol Biotechnol 81:515–522. doi:10.1007/s00253-008-1695-8. 18800183

