# Supplementary material for: Expressing Parasitoid Venom Protein VRF1 in an Entomopathogen Beauveria bassiana Enhances Virulence toward Cotton Bollworm Helicoverpa armigera
Source: Appl Environ Microbiol. 2023 Jun 5;89(6):e00705-23. doi: 10.1128/aem.00705-23 (PMC10304766; doi:10.1128/aem.00705-23)
Supplement: Supplemental file 1 — Supplemental material. Download aem.00705-23-s0001.pdf, PDF file, 0.3 MB [file aem.00705-23-s0001.pdf]

Table S1. Sequences of primers used in the study

Primer	Forward /Reverse	Sequence	Notes
Bb_sp	F	ATGGCTCCTTTTCTTCAAACC	Gene cloning of signal peptide of *Bbchit1*
	R	GGTTGCGCAGGTGTC	
VRF1	F	GCAGAAGTGATTAATAGAAGACAT	Gene cloning of *VRF1* (without signal peptide)
	R	AGCACTTGAGTCATTTATGAG	
mCherry	F	ATGGTGAGCAAGGGCGAGGAG	Gene cloning of *mCherry*
	R	TTACTTGTACAGCTCGTCCATGC	
L-pPK2	F	TCATATAACCAATTGCCCTCATC	Plasma linearization
	R	TCATGTATGTAGTGGGTGTGC	
R-Bb_sp	F	**GGCTGCAGGAATTCGATATC**ATGGCTCCTTTTCTTCAAACCA	Recombinant cloning for pPK2-Bar-EGFP-VRF1
	R	**TAATCACTTCTGC**GGTTGCGCAGGTGTCGGC	
R-VRF1	F	**CGCAACC**GCAGAAGTGATTAATAGAAGACATGTTACA	
	R	**TGCTCACCAT**AGCACTTGAGTCATTTATGAGATTTTC	
R-mCherry	F	**CTCAAGTGCT**ATGGTGAGCAAGGGCGAGG	
	R	**TCGACGGTATCGATAAGCTT**TTACTTGTACAGCTCGTCCATGCC	
gBVmt	F	AAGTATGCAAAGCATGCGGAG	Verification of cassette from *gpdA* promotor to *trpC* terminator
	R	ACATGCATTGCAGATGAGCTGTATC	
Q-Bb-actin	F	AGATTTGGCACCACACCTT	Semi-quantitative RT-PCR
	R	GGCAGCAGTCTGAATCTCCT	
Q-VRF1	F	GAACATTGTAGTTGAAGCCACA	
	R	ATAAGAGCCAGTACTATTTTCCT	
Ha_defensin	F	GAGAGACTCCTCCGTGTTGC	Quantitative RT-PCR
	R	CGTCGTTTCTTCGGAATCGC	
Ha_moricin1	F	TTCGGCTTAGTAGTTCTTGT	
	R	GTGGCCAGTGCCGATCGCAC	
Ha_moricin5	F	AACAACCGACCCCGCTTTC	
	R	TTCTTCATGGTGATTCTGGC	
Ha_lysozyme1	F	GAGTTGAGGAGGCAAGGGTT	
	R	ATACAGGCCGTAGTCTCGGG	
Ha_RpS3	F	ACGGAGTTTTCAAGGCGGAA	Quantitative RT-PCR (internal standard)
	R	GACTGCTCCGGGATGTTGAA	
P.S. : sequences in bold font are the overlaps for homologous recombinant cloning.			

Table S2. Comparison of virulence between WT and BbVRF1 against H. armigera larvae in laboratory conditions

	LT_{50} (days) (95% confidence interval)	LD_{50} ($\times 10^3$ conidia) (95% confidence interval)
Wild type	5.69 (3.52 – 6.63)	2.90 (2.27 – 3.60)
BbVRF1	4.79 (4.63 – 4.95)	1.23 (0.96 – 1.53)

LT_{50}: median lethal time at 1×10^4 conidia of injection treatment.

LD_{50}: median lethal dose at 8 days after the injection treatments (1×10^2, 1×10^3, 5×10^3, 1×10^4 and 2.5×10^4 conidia).

Table S3. Bioassay results of WT and BbVRF1 on *H. armigera* and *S. frugiperda* under gradient doses

Injection dose (number of conidia)	Number of larvae	Number of mortality			
		Ha.		*Sf.*	
		WT	BbVRF1	WT	BbVRF1
Control (1×PBS)	72	0	0	0	0
1.0×10^2	72	0	5	0	5
1.0×10^3	72	15	30	12	25
5.0×10^3	72	47	61	33	53
1.0×10^4	72	60	70	45	63
2.5×10^4	72	72	72	59	68

Table S4. Time-course bioassay results of WT and BbVRF1 on *H. armigera* and *S. frugiperda* after injection of 1×10^4 conidia

Days after injection	Number of cumulative mortality					
	Ha.			*Sf.*		
	Control	WT	BbVRF1	Control	WT	BbVRF1
1	0	0	0	0	0	0
2	0	0	0	0	0	0
3	0	0	0	0	0	0
4	0	0	7	0	0	0
5	0	18	48	0	0	14
6	0	49	65	0	9	50
7	0	54	70	0	22	62
8	0	66	72	0	45	63

Table S5. Comparison of virulence between WT and BbVRF1 against *S. frugiperda* larvae in laboratory conditions

	LT_{50} (days) (95% confidence interval)	LD_{50} ($\times 10^3$ conidia) (95% confidence interval)
Wild type	6.59 (6.32 – 6.98)	5.83 (4.47 – 7.55)
BbVRF1	4.81 (4.56 – 5.03)	1.69 (1.22 – 2.24)

LT_{50}: median lethal time at 1×10^4 conidia of injection treatment.

LD_{50}: median lethal dose at 8 days after the injection treatments (1×10^2, 1×10^3, 5×10^3, 1×10^4 and 2.5×10^4 conidia).
