# Supplementary material for: Expressing Parasitoid Venom Protein VRF1 in an Entomopathogen Beauveria bassiana Enhances Virulence toward Cotton Bollworm Helicoverpa armigera
Source: Appl Environ Microbiol. 2023 Jun 5;89(6):e00705-23. doi: 10.1128/aem.00705-23 (PMC10304766; doi:10.1128/aem.00705-23)
Supplement: Supplemental file 2 — Supplemental material. Download aem.00705-23-s0002.pdf, PDF file, 0.7 MB [file aem.00705-23-s0002.pdf]

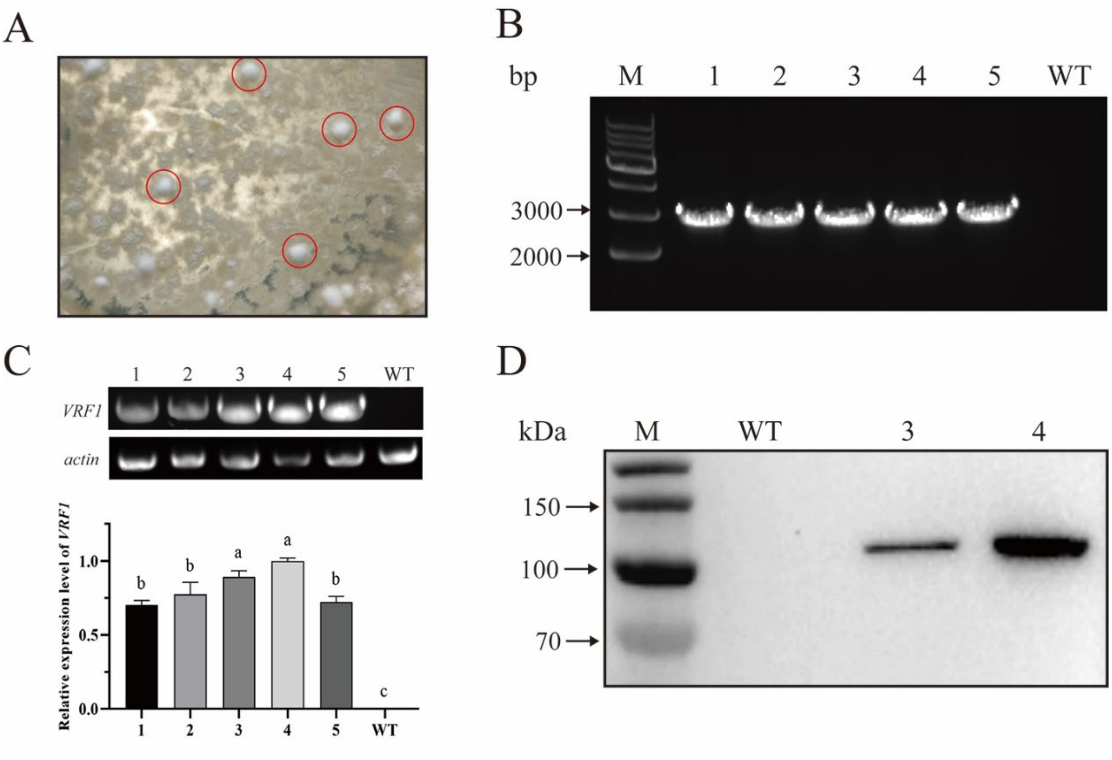

Figure S1. Screening of BbVRF1.

(A) Positive transformants on the PDA plate with phosphinothricin. (B) PCR detection for the presence of the fragment (cassette *gpdA* promotor + *Bbsp* + *VRF1* + *mCherry* + *trpC* terminator) in the genomic DNA of the transformants and wild-type (WT) strain (negative control). (C) Semi-quantitative RT-PCR analysis of *VRF1* in the cDNA of transformants and WT. The *actin* gene was used as an internal reference. (D) Western blot of the secretory venom protein rVRF1 in Sabouraud dextrose broth (SDB) of WT, candidate transformant 3 and transformant 4. M, marker.

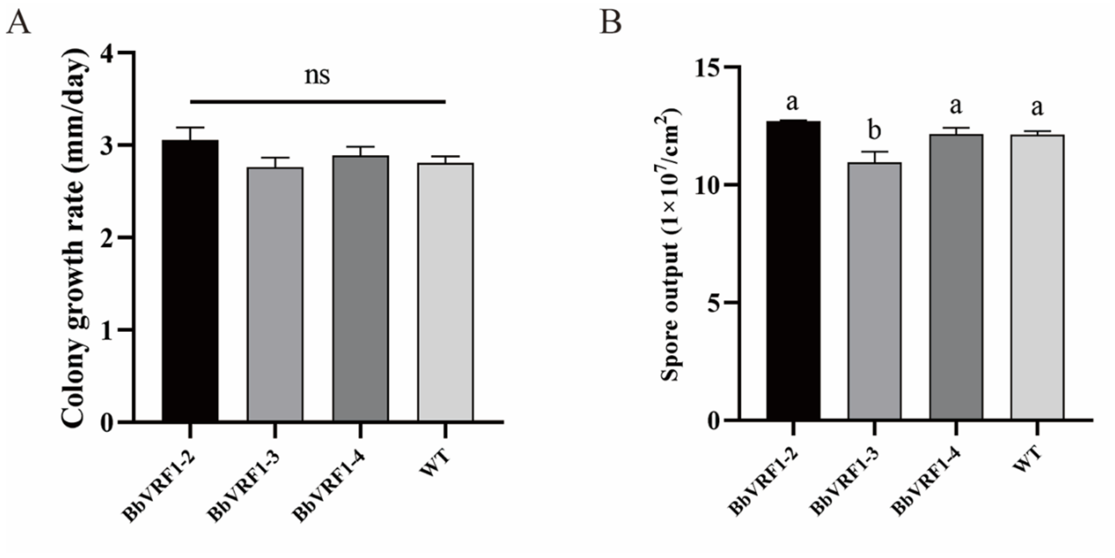

Figure S2. Comparisons of colony growth rate and spore output between transformants and WT

(A) The colony growth rate of candidate transformants (BbVRF1-2, BbVRF1-3 and BbVRF1-4) and wild type (WT). (B) The spore output of candidate transformants (BbVRF1-2, BbVRF1-3 and BbVRF1-4) and WT. Data are shown as means ± SD of five biological replicates. Ordinary one-way ANOVA with Tukey's multiple comparisons test was performed (p-value ≤ 0.05).

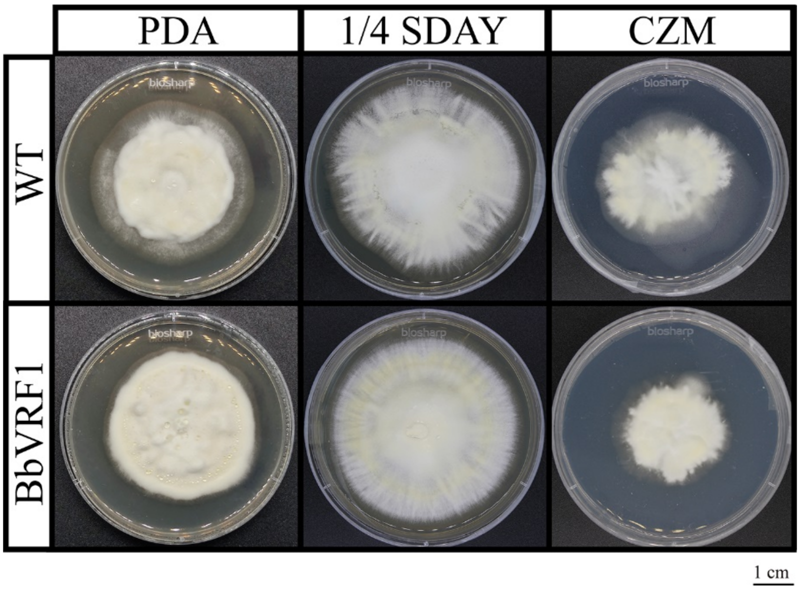

Figure S3. Phenotype of wild-type *B. bassiana* and transformant BbVRF1.

The suspensions of wild type (WT) and BbVRF1 were spotted on PDA, 1/4 SDAY, and CZM plates and then incubated at 26 °C for 20 days.

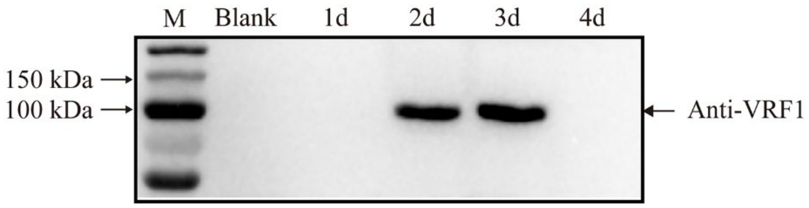

Figure S4. Western blot of VRF1 in the hemolymph of *H. armigera* infected by BbVRF1.

To verify the expression of VRF1 in the BbVRF1 infected *H. armigera*, the western blot of hemolymph was performed using VRF1 antibodies. The hemolymph of the *H. armigera* was collected at 1, 2, 3 and 4 days post-infection by BbVRF1, and hemocytes were removed by centrifugation.

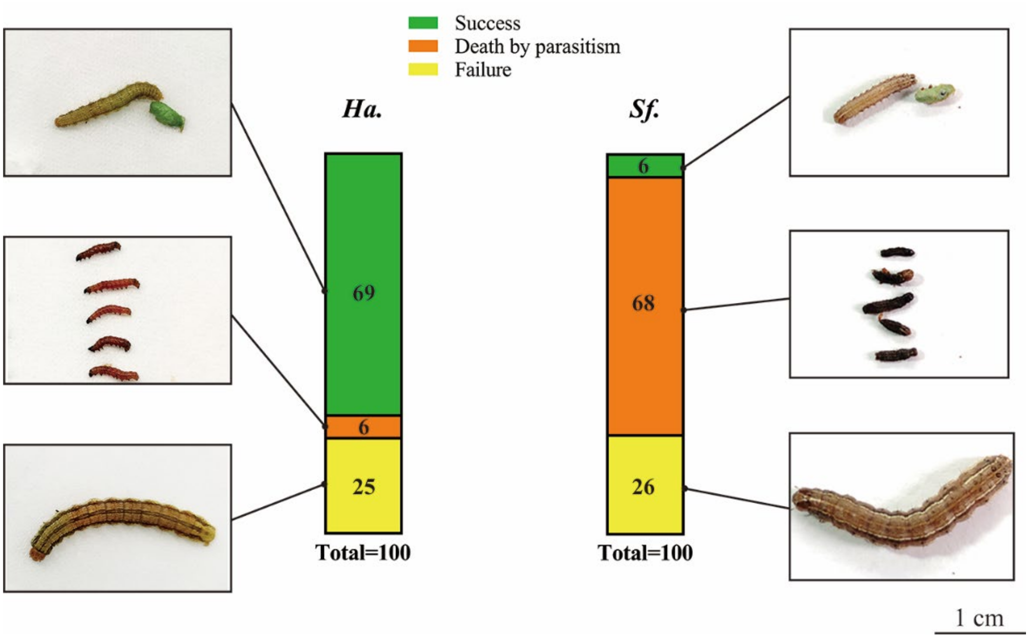

Figure S5. Parasitism ratios of *H. armigera* and *S. frugiperda* parasitized by *M. mediator.*

Parasitism ratios of *H. armigera* and *S. frugiperda* were counted after being parasitized by *M. mediator*. Larval hosts were divided into three types based on their phenotypes: success, death by parasitism, and failure. Success, wasp offspring developed normally and hosts were still alive. Death of parasitism, hosts died after being parasitized within 12 h without microbial infection. Failure, hosts developed normally after parasitized.
